# Fast detection of extrasynaptic GABA with a whole-cell sniffer

**DOI:** 10.3389/fncel.2014.00133

**Published:** 2014-05-15

**Authors:** Rasmus K. Christensen, Anders V. Petersen, Nicole Schmitt, Jean-François Perrier

**Affiliations:** ^1^Department of Neuroscience and Pharmacology, Faculty of Health and Medical Sciences, University of CopenhagenCopenhagen, Denmark; ^2^Department of Biomedical Sciences, Faculty of Health and Medical Sciences, University of CopenhagenCopenhagen, Denmark

**Keywords:** GABA, ambient, spillover, extrasynaptic, inhibition

## Abstract

Gamma-amino-butyric acid (GABA) is the main inhibitory transmitter of the brain. It operates by binding to specific receptors located both inside and outside synapses. The extrasynaptic receptors are activated by spillover from GABAergic synapses and by ambient GABA in the extracellular space. Ambient GABA is essential for adjusting the excitability of neurons. However, due to the lack of suitable methods, little is known about its dynamics. Here we describe a new technique that allows detection of GABA transients and measurement of the steady state GABA concentration with high spatial and temporal resolution. We used a human embryonic kidney (HEK) cell line that stably expresses GABA_A_ receptors composed of α1, β2, and γ2 subunits. We recorded from such a HEK cell with the whole-cell patch-clamp technique. The presence of GABA near the HEK cell generated a measurable electric current whose magnitude increased with concentration. A fraction of the current did not inactivate during prolonged exposition to GABA. This technique, which we refer to as a “sniffer” allows the measurement of ambient GABA concentration inside nervous tissue with a resolution of few tens of nanomolars. In addition, the sniffer detects variations in the extrasynaptic GABA concentration with millisecond time resolution. Pilot experiments demonstrate that the sniffer is able to report spillover of GABA induced by synaptic activation in real time. This is the first report on a GABA sensor that combines the ability to detect fast transients and to measure steady concentrations.

## INTRODUCTION

γ-Aminobutyric acid (GABA) is the main inhibitory neurotransmitter in the central nervous system. Its importance is illustrated by the severity of pathologies such as epilepsy, seizures, schizophrenia, or anxiety, which all have been linked to abnormal GABA functions (Ling et al., 1998; Feucht et al., 1999; Benes and Berretta, 2001; Wallace et al., 2001). GABA is synthesized by inhibitory interneurons throughout the central nervous system. Once released, GABA diffuses in the synaptic cleft and activates ionotropic and metabotropic receptors. GABA exerts its direct inhibitory effect by the hyperpolarization and the shunt mediated by ionotropic ligand gated Cl^-^ channels (Chance et al., 2002; Wong et al., 2003). GABA receptors are expressed subsynaptically and extrasynaptically in post-synaptic neurons (Ingham and McAlpine, 2005). The extrasynaptic receptors have particularly high affinity for GABA (Farrant and Nusser, 2005). They are activated by transient GABA overflow from the GABAegic synapses (Farrant and Nusser, 2005). In addition, extrasynaptic receptors are activated by ambient GABA continuously present in the extracellular space (Scanziani, 2000; Farrant and Nusser, 2005; Alle and Geiger, 2007; Bright et al., 2011). Extrasynaptic GABA receptors adjust the excitability of neurons (Otis et al., 1991; Salin and Prince, 1996; Bautista et al., 2010) and regulate the behavior of neuronal networks (Hamann et al., 2002). However, the dynamics of their activation remains unknown. Different studies of the estimates of the ambient GABA concentration in the brain range from ten nanomolars to a few micromolars (Juhasz et al., 1997; Maex and De Schutter, 1998; Huang et al., 2008; Numata et al., 2012). Indeed, the level of extrasynaptic GABA changes with functional state (Lerma et al., 1986; Rowley et al., 1995; Kennedy et al., 2002).

Measuring the variations in ambient GABA concentration is therefore essential for understanding the dynamics of signaling in the brain. So far microdialysis combined with high performance liquid chromatography has been used to measure GABA levels in nervous tissue (Lerma et al., 1986; Kennedy et al., 2002; de Groote and Linthorst, 2007; Vanini et al., 2011). This provides a static picture with time resolution of several minutes, far slower than the time scale of information processing in the brain. Another approach for detecting GABA consists in making an excised outside-out patch from a cell from the central nervous system with the assumption that it contains several GABA_A_ receptors (Isaacson et al., 1993). The isolation of GABA_A_ receptors requires pharmacological manipulations such as blocking GABA transporters and receptors for other neurotransmitters (Isaacson et al., 1993; Banks and Pearce, 2000; Liu et al., 2005). This technique allows the detection of GABA transients in the extracellular space. However, it is not suitable for measuring ambient GABA because the concentration depends on neuronal activity (Kekesi et al., 1997; de Groote and Linthorst, 2007; Vanini et al., 2011), which is obviously affected by blockade of receptors.

Here we report an innovative method for fast and sensitive detection of extracellular GABA in brain tissue. We used a human embryonic kidney (HEK) cell line that stably expresses GABA_A_ receptors that respond to GABA with a current that decays to a steady state. By recording one HEK cell with whole-cell patch-clamp technique, we obtained a sniffer that transduces GABA into a measurable current. We demonstrate that the sniffer can be transferred into slices from the central nervous system where it can measure the concentration of ambient GABA and detect spillover. To the best of our knowledge, this is the first GABA sensor that combines both, the ability to detect fast transients and to measure steady concentrations.

## MATERIAL AND METHODS

### GABA SNIFFER

Human embryonic kidney (HEK) cells 293 expressing GABA_A_ receptors (gift from NeuroSearch A/S, Ballerup, Denmark) were grown in T75 flasks (VWR, Herlev, Denmark) in full DMEM media (DMEM supplemented with 100 U/mL penicillin, 100 mg/ml streptomycin, and 10% fetal calf serum; (Sigma-Aldrich, Copenhagen, Denmark) at 37°C, in a humidified atmosphere with 5% CO_2_. Confluent cells were rinsed with phosphate buffered saline (in-house, University of Copenhagen), trypsinized (1%) and plated on 9 mm coverslips (VWR). Cells were allowed to settle on coverslips for a minimum of 30 min before being transferred to a recording chamber, where they were positioned next to a spinal cord slice.

**FIGURE 1:**
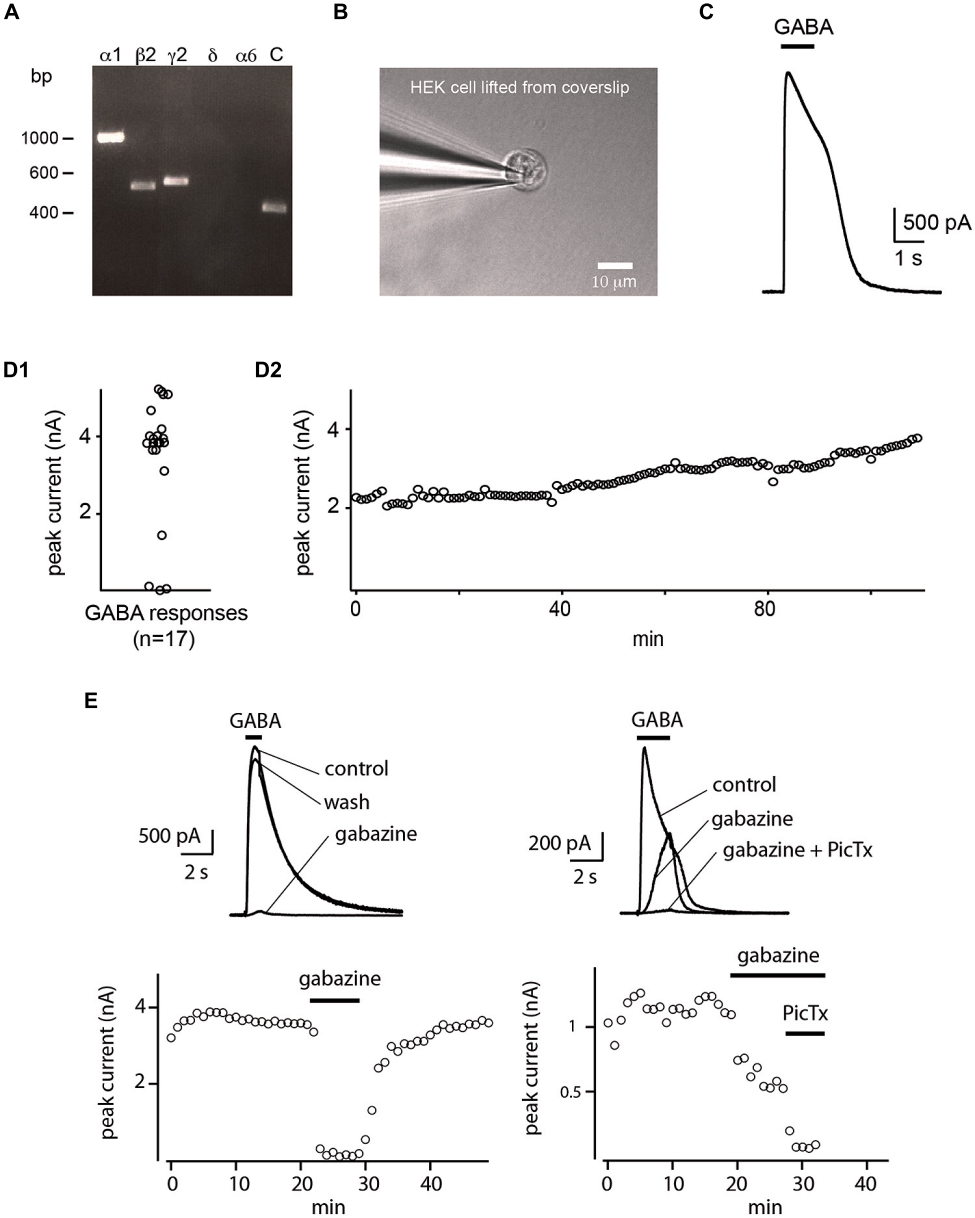
**Whole-cell sniffer with GABA_A_ receptors. (A)** RT-PCR verifying the expression of GABA_A_ receptors composed of α1β2γ2 subunits in HEK cells. Primers for α6 and δ subunits were included as control. Expression of hGAPDH served as control **(C)** of cDNA quality. **(B)** Whole-cell recording of a HEK cell detached from the coverslip. **(C)** A single puff of GABA evoked a large outward current. **(D1)** Distribution of the amplitude of the evoked currents. 82% (14/17) of the HEK cells tested with GABA responded with large outward currents. **(D2)** Amplitude of the current evoked by repetitive GABA puffs applied every 60 s. The response was stable over hours (*n* = 2). **(E)** Response of two sniffers to GABA. The evoked current was suppressed by gabazine (10 μM) or by further addition of picrotoxin (50 μM).

HEK cells were recorded in whole-cell configuration in voltage-clamp mode. The recording pipette was mounted on a 3-axes motorized micromanipulator (Luigs and Neumann, Ratingen, Germany). Their membrane potential was held at 0 mV. The sensitivity of HEK cells to GABA was systematically tested by measuring the current evoked by a puff of GABA (1 mM; Sigma-Aldrich; see **Figure 1**).

### REVERSE TRANSCRIPTION (RT)-PCR

Total RNA was prepared from HEK293 cells constitutively expressing GABA_A_ receptors using the RNeasy Mini Kit (Qiagen, Hilden, Germany) according to the manufacturer’s instructions. The RNA was reverse-transcribed using a poly-T primer (5′-T24–V-N-3′) and Superscript^TM^ III according to the manufacturer’s instructions (Invitrogen, Carlsbad, CA, USA). Controls were performed in the absence of template. The RT products were PCR amplified using PfuUltra II polymerase (Stratagene) and oligodeoxynucleotides primers for GABA_A_ subunits (**Table 1**). The cycling conditions were 2 min at 94°C; 25 cycles at 94°C for 30 s, annealing temperature differing between primer pairs (see **Table 1**) for 40 s, 72°C for 30 s; 72°C for 5 min. Amplified products were analyzed using 1% agarose gels. The PCR products were sequenced for identity confirmation (Macrogen Inc., Seoul, Republic of Korea).

**Table 1:** Primers and conditions for reverse-transcription PCR.

Subunit	GenBank Acc. No.	Primer Sequence (5’-3’);T_m_ [°C]	T_ann_[°C]	amplicon [bp]	Ref
α1	NM_001127644.1	F: TGAGCACACTGACTGGAAGAAGC; 62,4	55	999	Maddox et al. (2004)
		R: GAACCACACTTTTGCCATCCC; 59,8
α6	NM_000811.2	F: TGATGGTCAGTAAAATCTGGACGC; 61,0	55	507	Maddox et al. (2004)
		R: AAACAGTTCTTGCTGGGACGG-3’; 59,8
β2	NM_021911.2	F: TGCCTGATACCTATTTCCTGAACG-3’; 61,0	55	488	Maddox et al. (2004)
		R: GATTCCTAATGCCACCCTTGC-3’; 59,8
Δ	NM_000815.4	F: AGGACATCGTCTACTACTGGTCGGAGAG-3’; 64,4	55	439	Maddox et al. (2004)
		R: TCGGCGTTGAAATGAGCAAAGG-3’; 60,3
γ 2	NM_198904.2	F: TGCACACTCATTGTCGTCCTATCCTGG-3’; 66,5	60	524	Maddox et al. (2004)
		R: TTAAACAGGCAGAAGGCAGTGGGG-3’; 64,4
hGAPDH	NM_017008	F: cacccatggcaaattccatg; 57.3	55	372	Einarsen et al. (2009)
		R: catgagtccttccacgatac; 57.3

*Forward (F) and reverse (R) primers used for RT-PCR of human GABA_A_ subunits. Expected amplicon sizes refer to cDNA according to GenBank accession numbers.*

### SLICE PREPARATION

Adult turtles (*Trachemys scripta elegans*) were anesthetized by intravenous injection of propofol (0.3 ml/100 g; Propolipid Frenesius Kabi, Sweden) and killed by decapitation. The surgical procedures complied with Danish legislation. The spinal cord was removed after intra-cardiac perfusion of a high Mg^2+^ solution (in mM: 120 NaCl, 5 KCl, 15 NaHCO_3_, 20 Glucose, 20 MgCl_2_, 3 CaCl_2_). All experiments were performed at room temperature (20–22°C).

The lumbar enlargement (D8-S2) was cut into slices (300–1500 μm thick) with a vibratome (MicroM slicer HM 650 V; Microm International GmbH, Germany) equipped with cooling unit CU65 set at 2°C. For some experiments, a dorsal root filament was left in continuity with the slice and mounted on a suction electrode connected to a stimulator. Slices were continuously perfused with Ringer’s solution (in mM: 120 NaCl, 5 KCl, 15 NaHCO_3_, 20 Glucose, 2 MgCl_2_, 3 CaCl_2_) saturated with 98% O_2_ and 2% CO_2_ to obtain a pH of 7.6.

### PATCH-CLAMP RECORDING

Visually guided patch-clamp recordings were performed in whole-cell configuration with a Multiclamp 700B amplifier (Molecular Devices, USA) in voltage-clamp mode. Cells were visualized by means of a BW51WI microscope (Olympus, Japan) equipped with an oblique illumination condenser or with a differential interference contrast system. The pipette solution (in mM: 122 K-gluconate, 2.5 MgCl_2_, 5.6 Mg-gluconate, 5 K-HEPES, 5 H-HEPES, 5 Na_2_ATP, 1 EGTA, 2.5 biocytine, HCl to adjust the pH to 7.4) contained the fluorescent dye Alexa 488 (250 μM, Sigma-Aldrich). Electrodes had an input resistance ranging from 4 to 8 MΩ. Recordings were sampled at 10–20 kHz and treated offline with a Bessel filter with a cutoff frequency set at 400 Hz. Data were sampled with a 16-bit analog-to-digital converter (DIGIDATA 1440; Molecular Devices, USA) and displayed by means of Clampex 10.2 software (Molecular Devices, USA). Data are presented without liquid-junction potential correction unless specified.

### FOCAL APPLICATION OF DRUGS

Electrodes made from borosilicate capillaries with tip diameter <1.5 μm (G150F-3; Warner Instruments, USA) were filled either with GABA (1 mM in Ringer’s solution) or glutamate (1 mM in Ringer’s solution; Sigma-Aldrich). Drugs were puff applied at 14–35 Pa by a homemade time-controlled pressure device.

### GLUTAMATE UNCAGING

Caged glutamate (MNI-caged-L-glutamate; 10 μM; Tocris Bioscience, UK) was added to the bath. Glutamate was uncaged at different points of interest (POI) by means of an UGA 40 scanning system generating light spots with a diameter of ~5 μm coupled to a 375 nm laser (Rapp OptoElectronic GmbH, Wedel, Germany).

### STATISTICAL ANALYSIS

Data were analyzed by means of IGOR Pro 6.22 (Wavemetrics, Tigard, OR, USA) and Matlab (MathWorks; Natick, MA, USA). The normality of distribution of each sample was tested with One Sample Kolmogorov–Smirnov test. Non-parametric tests were used when Gaussian distribution could not be approximated. Data are represented as median ± standard deviation. Mean ± standard error (SE) is used when indicated.

### DRUGS

The following drugs were used: glutamate (Sigma-Aldrich), γ-Aminobutyric acid (Sigma-Aldrich), MNI-caged-L-glutamate (Tocris Bioscience, Abingdon, UK), Tetrodotoxin (TTX; Alomone Labs, Jerusalem, Israel), SR 95531 hydrobromide (gabazine, Tocris Bioscience), Picrotoxin (Tocris Bioscience).

## RESULTS

### HEK293 CELL LINE PROPERTIES

We verified that the HEK cell line expressed α1, β2, and γ2 subunits of the GABA_A_ receptor by performing non-quantitative RT-PCR using primers described by Maddox et al. (2004). Amplicons of the expected molecular sizes were obtained for α1, β2, and γ2 subunits. We used primer pairs for α6 and δ subunits as negative controls, and as expected, none of these subunits were present in the cell line (**Figure 1A**). As additional negative control, we performed the reactions in the absence of template DNA (data not shown). The expression of the housekeeping gene GAPDH served as control of the quality of the prepared cDNA. The identity of PCR-products was confirmed by sequencing.

**FIGURE 2:**
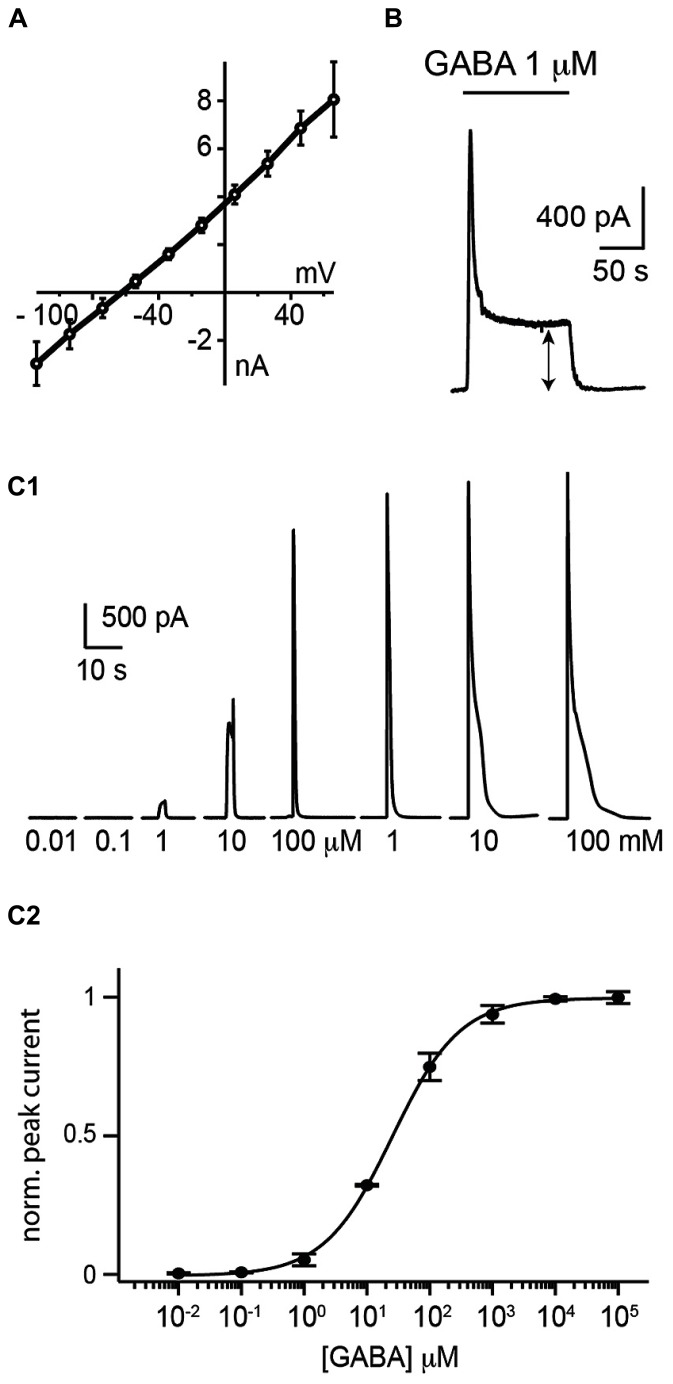
**Electrophysiological properties of the whole-cell sniffer. (A)** I/V plot of sniffer responses to GABA (1 mM) from 7 cells clamped from -120 to 80 mV. Values were corrected for liquid junction potential. The average reversal potential (E_GABA_) was of -67 mV. **(B)** Response of a sniffer to a prolonged exposure of GABA. The sniffer exhibited a non-inactivating component (double arrow). **(C1)** Responses of a sniffer to 1 s puff of increasing GABA concentrations (from 0.01 μM to 100 mM). **(C2)** Hill plot of dose–responses relationships of peak currents (*r* = 0.99994). Responses were normalized to the fitted *I*_max_ value. The average EC_50_ for the peak response was 26.3 ± 3.0 μM (mean ± SE; *n* = 3).

HEK 293 cells were plated on coverslips and moved to a recording chamber continuously perfused with Ringer’s solution. We measured how the cells responded to saturating concentrations of GABA by recording them with the patch-clamp technique in whole-cell configuration in voltage-clamp mode (holding potential *V*_h _= 0 mV; **Figure 1B**). In our experimental conditions, we calculated a reversal potential for chloride ions of -71.8 mV. We found that an 1 s puff (14–35 Pa) of high concentration of GABA (1 mM) applied through a glass pipette positioned at a distance of 20–30 μm, induced a strong outward current (median amplitude: 3941 ± 961 pA; *n* = 17; **Figures 1C,D1**) with a reversal potential of –67 ± 14 mV (*n* = 7; **Figure 2A**). The conductance of single α1β2γ2 GABA_A_ receptor is in the range of 19–26 pS. Using Ohm’s low, we approximated that each sniffer contains 3000–4200 GABA_A_ receptors. The rise time of the current estimated as the duration from 20 to 80% of the peak current varied from 11.4 to 40.3 ms, corresponding to a median rate of rise of 114 ± 96 pA/ms. The majority of cells responded to GABA since only 3/17 (17.6 %) remained silent during puff application. The evoked responses were remarkably stable. Consecutive puff applications of GABA over more than 1 h evoked responses that did not decrease in amplitude (**Figure 1D2**; *n* = 2). The evoked currents were reversibly inhibited by gabazine (10 μM; two out of four trials; **Figure 1E**). In some instances, gabazine alone was not sufficient to eliminate the evoked current. However, after addition of the GABA_A_ receptor blocker picrotoxin (50 μM) the response was always abolished (**Figure 1E**; *n* = 3). To further characterize the sniffer, we established a concentration-response relationship by puffing different concentrations of GABA. We found that the amplitude of the current increased with the concentration of GABA (**Figure 2C1**). By fitting a Hill equation to the normalized amplitude of the current as a function of the GABA concentration (*r* = 0.99994), we determined a half maximal effective concentration (EC_50_) of 26.3 ± 5.2 μM with Hill coefficient of 0.8 ± 0.02 (*n* = 3; **Figure 2C2**).

During prolonged applications of GABA, the sniffer exhibited fast desensitization with decay time constants (*t*) ranging from 0.8 to 4.8 s. However, a significant component corresponding to approximately one fifth (18 ± 12%; *n* = 5) of the maximal response did not desensitize (**Figure 2B**). The remaining tonic current had an amplitude that increased with the concentration of GABA. Since the sniffer generated a persistent current, we investigated if this enabled us to estimate the ambient concentration of GABA inside nervous tissue.

### DETECTION OF AMBIENT GABA CONCENTRATION IN A SLICE PREPARATION

**FIGURE 3:**
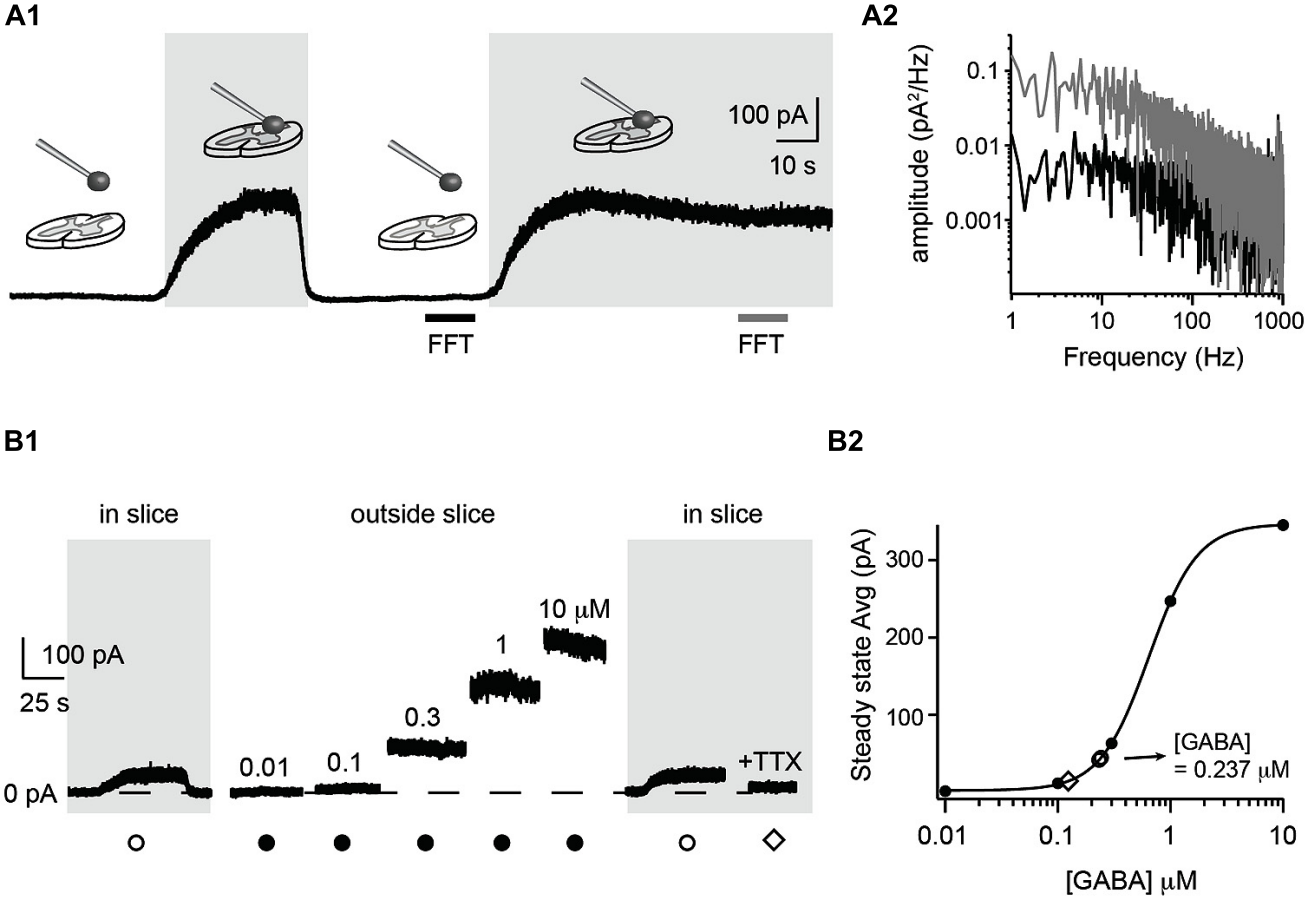
**Detection and quantification of ambient GABA in a slice preparation. (A1)** Response of a sniffer at different positions above a slice preparation from the spinal cord. Positioning the sniffer at the slice surface evoked a persistent outward current and increased the noise of the recording. Both current and noise disappeared when the sniffer was moved 500 μm above the slice. The gray and black horizontal bars indicate the time intervals used for Fast Fourier Transformation (FFT) analysis in **A2**. **(A2)** Power spectrum densities of the current measured by the sniffer far from the slice (black) and on the surface (gray). The frequencies between 1 and 1000 Hz were stronger when the sniffer was just above the surface of the slice. **(B1)** Response of a sniffer positioned in a slice (gray background) or outside the slice (white background). The responses far from the slice were evoked by applying incremental GABA concentrations in the extracellular medium. TTX (50 nM) was added in the end of the experiment. **(B2)** Hill plot of concentration–response relationship of steady state currents evoked by GABA in the extracellular medium. The two open circles represent the measurements from the surface of the slice before and after calibration. The average concentration of GABA obtained from the curve was of 0.237 μM. The open diamond represents the measurement obtained in TTX.

To measure the extracellular GABA concentration in the central nervous system, we positioned a coverslip with HEK cells near a slice preparation from the spinal cord. We recorded one HEK cell in whole-cell configuration and moved it to the surface of the spinal cord by means of a motorized micromanipulator (**Figure 3A1**). The membrane resistance was continuously monitored. When lowering the sniffer to the surface of the slice, we often recorded large steady-state outward currents (up to ~500 pA) and a concomitant increase in electrical noise (**Figure 3A1**). A fast Fourier transform analysis revealed that the frequencies below 500 Hz were strengthened (**Figure 3A2**). This probably reflects the stochastic fluctuations in the GABA_A_ receptor channels opening and closing (Mtchedlishvili and Kapur, 2006) which generate a noise in the band from 0 to 200 Hz (Kaneda et al., 1995; Brickley et al., 1996; **Figure 3A2**). The amplitude of the current varied with the distance between the slice surface and the sniffer. The first signs of tonic currents were usually detected at a distance of approximately 50 μm above the slice and increased to a maximum when touching the surface of the slice tissue. At this stage, the sniffer cell was slowly moved down in the tissue at a speed of around 1 μm/s. The progression was stopped as soon as the membrane resistance decreased more than 10%. Using this procedure it was possible to reach depths of 80 μm without losing the recording. The sniffer also detected GABA inside the slice. The currents recorded were not stronger than just above the surface. To quantify the actual concentration of GABA in the slice, we withdrew the sniffer and positioned it at a distance where no current was detected (i.e., at least 500 μm above the slice). We then applied increasing concentrations of GABA (ranging from 0.01 to 10 μM) in the bath and measured the evoked steady state currents (**Figure 3B1**). By fitting a Hill equation to the amplitude of the steady-state current as function of the GABA concentration, we obtained a standard concentration-response curve (*r* = 0.9998; **Figure 3B2**). We used the curve to estimate the GABA concentration in relation to measured currents. Finally, a second measurement was performed at the surface of the slice to confirm the value obtained with the first measurement. In the example illustrated in **Figure 3B1**, we measured a current of 41.9 pA in the slice. This corresponded to an average concentration of 0.232 μM on the Hill plot (**Figure 3B2**). Repeating the procedure after calibration confirmed our first estimation (44.8 pA corresponding to 0.242 μM). We calibrated the sniffer for the experiments performed in two slices. The sniffer detected ambient GABA in both slices, with a mean concentration of 0.176 μM. In slices made from 2 of the 11 animals, we did not detect a tonic GABA signal. The sensitivity of our equipment allows us to measure currents as low as 10 pA, corresponding to a GABA concentration of 0.1 μM. The lack of tonic current indicates that the ambient concentration of GABA is below this value. Next, we tested if the level of ambient GABA depended on neuronal activity. For this purpose we added the sodium ion channel blocker tetrodotoxin (TTX; 10 μM; *n* = 1) to the extracellular medium. In the example of **Figure 3**, the steady state current of the sniffer dropped to 14.6 pA, corresponding to a GABA concentration of 0.124 μM (**Figures 2** and **3B1**). This test, which confirms that the tonic current recorded in the sniffer is not caused by an artifact, suggests that a substantial fraction of the ambient GABA has a neuronal origin. However, more systematic experiments are required to confirm this preliminary observation.

### DETECTION OF GABA TRANSIENTS IN A SLICE PREPARATION

**FIGURE 4:**
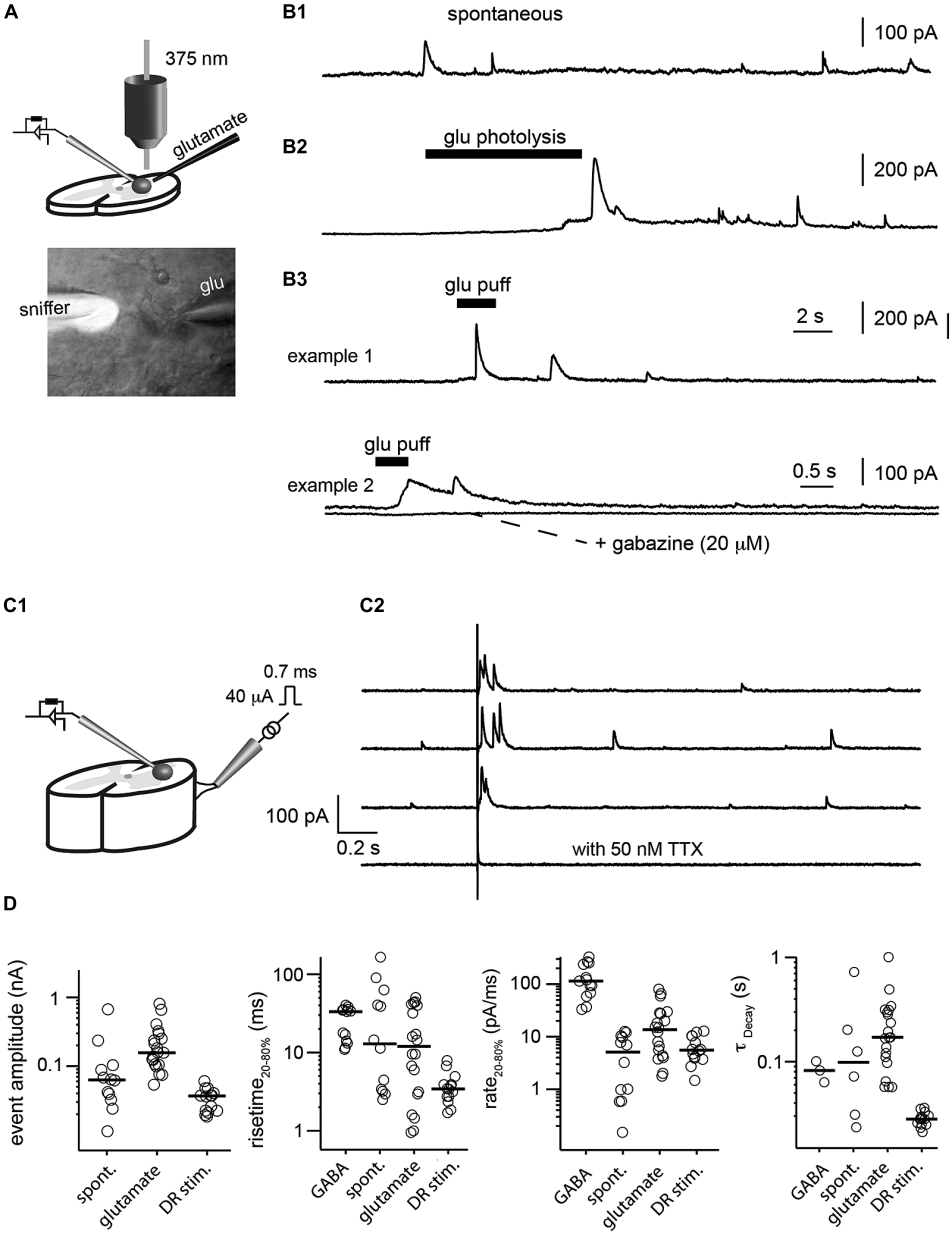
**Detection of GABA transients in a slice preparation. (A)** Illustration of the experimental setup. GABA release was evoked either by puffing glutamate or uncaging glutamate with a 375 nm laser. The lower picture illustrates a sniffer filled with the fluorophore Alexa 488 (white) placed inside a slice from the spinal cord. The pipette coming from the right was used to puff glutamate (glu). **(B)** Examples of transient events recorded in the sniffer. **(B1)** Spontaneous events. **(B2)** Events evoked by glutamate uncaging. **(B3)** Events evoked by glutamate puff. The responses were abolished after addition of the GABA_A_ receptor antagonist gabazine (20 μM). **(C1)** Scheme of the preparation. Slice from the spinal cord with a dorsal root filament stimulated with a suction electrode. Sniffer positioned in the slice. **(C2)** Response of the sniffer to single shocks applied on the dorsal root filament. Dorsal root stimulation evoked transient release of GABA. The responses were abolished after addition of TTX (50 nM). **(D)** Characteristics of the different types of events recorded by the sniffer (evoked by GABA, spontaneous, evoked by glutamate or dorsal root stimulation). From left to right: amplitude; rise time of the rising phase (from 20 to 80% of peak); rate of rise calculated as current from 20 to 80% of peak divided by rise time from 20 to 80% of the peak; time constant (τ) of the of the decay estimated by fitting a single exponential function.

When the sniffer was positioned inside the slice, we sometimes recorded spontaneous transient outward currents (**Figure 4B1**). They had a rise time of 12.9 ± 49 ms (measured from 20 to 80% of the maximal amplitude) and decayed with a time constant of 98 ± 267 ms (**Figure 4D**). Their rate of rise was 4.2 ± 3.5 pA/ms. These observations showed that extracellular GABA concentration varied phasically. Next, we evoked GABA release in the extrasynaptic space, either by puffing glutamate (1 mM) through a glass pipette (**Figures 4A,B3**) or by photolysing caged-glutamate in the vicinity of the sniffer cell (**Figures 4A,B2**). Both techniques evoked outward currents in the sniffer demonstrating that glutamate induces GABA release in the extrasynaptic compartment. The events evoked by puffing glutamate had rise times ranging from 0.6 to 65 ms (median 12.0 ± 18 ms) and a median rate of rise of 13.6 ± 22.8 pA/ms. The events induced by uncaging glutamate were slightly slower (4.2 ± 3.5 pA/ms). Since the rate of rise of events evoked by saturating concentrations of GABA were one order of magnitude faster (114.1 ± 96.7 pA/ms; **Figure 1C**), we can assume that the intrinsic properties of the sniffer were not a limiting factor for detecting transients. Our observations suggest instead that the measured transients reflected the actual local changes in GABA concentration that occurred near the sniffer. To confirm that the phasic events reflected variations in GABA concentrations, we added gabazine (20 μM) or picrotoxin (50 μM) to the extracellular medium. As expected, it eliminated all the events (**Figure 4B3**).

Next, we tested if the sniffer was sensitive enough for detecting release of GABA evoked by synaptic activation. For this purpose, we used a spinal cord preparation in which a dorsal root filament was left intact. We stimulated primary afferents by means of a suction electrode (**Figure 4C1**). A single electrical chock (40 μA, 700 μs) evoked transient outward currents in a sniffer positioned 80 μm below the surface of the dorsal horn (*n* = 3; **Figure 4C2**). The response was abolished after blockade of voltage sensitive sodium channels with tetrodotoxin (50 nM; **Figure 4C2**). This result suggests that the activity of sensory afferents induces a release of GABA in the extrasynaptic space and demonstrates that our sniffer is sensitive enough to detect it.

## DISCUSSION

We have demonstrated a novel method for measuring the ambient GABA and detecting variations in concentrations during neuronal activity. Our approach has several important advantages. First, the sniffer detects GABA with a temporal resolution in the order of 1 ms. In comparison, the detection rates achieved with other techniques such as microdialysis combined with HPLC or liquid chromatography–tandem mass spectrometry after butylation are on the order of minutes (Maddox et al., 2004; de Groote and Linthorst, 2007; Vanini et al., 2011). When saturating GABA concentrations were applied, the rates of rise of the evoked responses were significantly faster than the currents measured in slices. This suggests that we were able to detect local GABA concentration changes in real time. To our knowledge, this has never been reported before. Second, the sensitivity of the sniffer is reasonable since we were able to detect responses in the sniffer with ambient GABA concentrations as low as 0.1 μM. In excised outside-out patch made from central neurons, few receptors are present and it is not possible to establish a concentration–response relationship (Isaacson et al., 1993; Banks and Pearce, 2000; Gao and Van Den Pol, 2000; Liu et al., 2005). Third, the measurements of ambient GABA were reproducible. Moving the sniffer up and down a slice gave similar results. When the sniffer was calibrated it was possible to obtain accurate measurements of GABA concentration. Fourth, the sniffer allows detection of GABA without any pharmacological intervention. Detecting GABA with outside-out patch made from central neurons requires the blockade of receptors from other neurotransmitters such as glutamate, glycine, or acetylcholine (Isaacson et al., 1993; Liu et al., 2005). Employing the respective inhibitors will in turn affect the activity of the neuronal network. Ambient GABA and spillover are commonly studied with the help of pharmacological agents that isolate extrasynaptic receptors (Nusser and Mody, 2002) or block of GABA uptake mechanisms (Isaacson et al., 1993; Scanziani, 2000; Song et al., 2011). Our sensor is sufficiently sensitive to measure ambient GABA and to detect release in the extrasynaptic space with normal extracellular medium. Fifth, the sniffer has a small size (diameter below 10 μm). This allows for GABA detection in the proximity of GABA sources (e.g., synapses). In contrast, microdialysis probes have a diameter of more than a millimeter (Reynolds et al., 1999) and are not adapted for local detection of transmitters.

The method we developed also has limitations. Importantly, we cannot use the sniffer for recording deep in brain tissue. During our trials, we managed to position the sniffer down to 80 μm below the surface of slices. Attempts of moving deeper resulted in irreversible damages. However, we performed our experiments in slices from an adult vertebrate, which is heavily myelinated. It is likely that trials performed with embryonic or neonate nervous tissue containing little myelin would allow deeper recordings. Another disadvantage lies in the fact that the sniffer has to be calibrated for each experiment if one wants to quantify the concentration of GABA. This requires withdrawing the sniffer from the tissue before applying series of GABA concentrations in the bath. This procedure is difficult when the sniffer has been in the slice for several minutes. The seal between the recording electrode and the HEK cell often breaks at this stage. Finally, the sensitivity of our sniffer allowed us to detect concentrations in the order of 100 nM (see **Figure 3B**). This is not the most sensitive method available for measuring GABA concentration. Studies using microdialysis combined with HPLC reported concentrations as low as 1–10 nM (Rowley et al., 1995; Rea et al., 2005).

### INTERPRETATION OF RECORDINGS

The local concentration of GABA decays as a function of the distance between the source and the probe used to detect it. Diffusion of GABA in the extrasynaptic space also affects the time course of GABA transients: the more remote the source, the slower the kinetic (Allen, 1997). During our experiments, we observed transient events with slow rise times (>10 ms) compared to fast IPSCs (<1 ms) measured in post-synaptic neurons (Korshoej et al., 2010; Xie and Manis, 2013). This observation can be interpreted in two ways: (1) the source of GABA was distant from the sniffer; (2) The release of GABA occurs through a slow process, such as diffusion through cation channels (Song et al., 2011) or via the reversal of a GABA transporter (Reynolds et al., 1999). It is unlikely that the intrinsic properties of the sniffer contributed to shaping of transient events because it could react to high concentrations of GABA with much faster kinetics (~100 pA/ms) than the events detected in the tissue (~10 pA/ms).

### PERSPECTIVE

The presence of an ambient GABA concentration in the extracellular space of the brain is well accepted (Farrant and Nusser, 2005). However, the origin of GABA remains controversial. Some studies suggest that most of the ambient GABA originates from neuronal vesicular release (Brickley et al., 1996). Other publications indicate that the extracellular GABA has a non-vesicular source (Wall and Usowicz, 1997; Rossi et al., 2003). Recent investigations proposed that the extracellular GABA could also have a glial origin (Lee et al., 2010; Le Meur et al., 2012). Our technique could prove useful for identifying the cells that release GABA in the extrasynaptic space. Another challenge will be to apply this technique *in vivo*. Even though it appears technically challenging, we believe that it is feasible.

## AUTHOR CONTRIBUTIONS

Rasmus K. Christensen and Jean-François Perrier, conception and design of research. Nicole Schmitt, supervision of molecular biology. Rasmus K. Christensen and Anders V. Petersen, performed experiments. Rasmus K. Christensen analyzed data. Rasmus K. Christensen and Jean-François Perrier interpreted results of experiments. Rasmus K. Christensen and Jean-François Perrier wrote the manuscript. Rasmus K. Christensen, Anders V. Petersen, Nicole Schmitt and Jean-François Perrier approved final version of manuscript.

## Conflict of Interest Statement

The authors declare that the research was conducted in the absence of any commercial or financial relationships that could be construed as a potential conflict of interest.
